# Quantitative morphological transformation of vascular bundles in the culm of moso bamboo (*Phyllostachys pubescens*)

**DOI:** 10.1371/journal.pone.0290732

**Published:** 2023-09-21

**Authors:** Taku Tsuyama, Kensei Hamai, Yoshio Kijidani, Junji Sugiyama

**Affiliations:** 1 Faculty of Agriculture, University of Miyazaki, Miyazaki, Japan; 2 Graduate School of Agriculture, Kyoto University, Kyoto, Japan; Universidade Federal de Alfenas, BRAZIL

## Abstract

Vascular bundles of bamboo are determinants for mechanical properties of bamboo material and for physiological properties of living bamboo. The morphology of vascular bundles reflecting mechanical and physiological functions differs not only within internode tissue but also among different internodes in the culm. Although the distribution of vascular bundle fibers has received much attention, quantitative evaluation of the morphological transformation of vascular bundles associated with spatial distribution patterns has been limited. In this study deep learning models were used to determine quantitative changes in the distribution and morphology of vascular bundles in the culms of moso bamboo (*Phyllostachys pubescens*). A precise model for extracting vascular bundles from cross-sectional images was constructed using the U-Net model. Analyses of extracted vascular bundles from different internodes showed significant changes in vascular bundle distribution and morphology among internodes. Vascular bundles in lower internodes showed outer relative position and larger area than those in upper internodes. Aspect ratio and eccentricity indicate that vascular bundles in internodes near the base have more elliptical morphology, with a long axis in the radial direction. The variational autoencoder model using extracted vascular bundles enabled simulation of the morphological transformation of vascular bundles along with radial direction. These deep learning models enabled highly accurate quantification of vascular bundle morphologies, and will contribute to a further understanding of bamboo development as well as evaluation of the mechanical and physiological properties of bamboo.

## Introduction

Bamboo is known for its very fast growth and can grow to around 15 m in 3–4 months with no secondary growth [1, 2]. Based on this property, it is recognized as an important renewable resource for building a sustainable society [3]. For advanced utilization of bamboo, precise understanding of the tissue structures of bamboo culms is required.

Bamboo culms are hollow, encompassing a space called the central pith cavity. Bamboo culms have nodes and internodes, lengths of which change within a culm [1, 4, 5]. Mathematical simulations have shown that the presence of nodes and changing patterns of internode length effectively make the culm mechanically stronger [6]. The mechanical performance of the bamboo is generally considered to improve over time, throughout the period up to the third- and fourth-year culm [7]. The modulus of elasticity of the culm 105 days after shooting is almost stationary, but it then continues to increase until the ninth year of the culm [8]. This increase is considered to be largely the results of increasing density, which is caused by thickening of the cell wall of the vascular bundle fibers [2, 9].

The tissue of the culm has a cortical layer inside the epidermis and ground parenchyma, throughout which vascular bundles are scattered. Vascular bundle fibers with a thick secondary cell wall surround the protoxylem and metaxylem vessels, as well as the phloem. In bamboo, parenchyma cells of ground tissue as well as vascular bundle fibers form multi-layered cell walls [2, 10, 11]. Together with this development, various structures of hemicelluloses and lignin deposit in multiple cell wall layers with different angles of cellulose microfibril [2, 5, 10, 12]. After growth by elongation, these cell wall formations last for approximately 3–5 years, or even up to 9 years [2, 8].

The transverse sections of bamboo culms exhibit characteristic vascular bundle distributions along a radial direction [7, 13]. The mechanical strength of bamboo is as great as that of wood—or even higher [8, 14]. This strength can be derived from the distribution patterns of the vascular bundles of bamboo culms. By mathematical modeling, Sato et al. showed that the distribution pattern of vascular bundle fibers being dense on the outer side and sparse on the inner side effectively increases bending stiffness compared with a uniform distribution [15]. Some investigations have reported that the fraction of vascular bundle fibers increases linearly from the pith cavity to the epidermis side of the transverse section [16], whereas others have reported a parabolic increase [17, 18]. Sato et al. proposed a quadratic model, which showed that the effective distribution of vascular bundles fiber varies with the average volume fraction of the entire transverse section of each internode [15]. Other studies found that exponential function was the best to depict the radial distribution of the fiber volume fraction [18, 19], which is consistent with the elastic modulus distribution in the radial direction of the culm wall [20].

The morphology of the vascular bundles in transverse section also differs between the epidermal and pith sides [7, 13]. The epidermal side consists solely of thick-walled fiber cells, whereas the inner side shows the presence of the large phloem and metaxylem vessels. Differences in vascular bundle morphology may lead to differences in the size or the arrangement of the cells comprising the vascular bundles, which may contribute to both the mechanical properties and physiological functions of bamboo culms. A previous study suggests that the fraction of vascular bundle fibers is responsible for its tensile strength and the modulus of elasticity [21]. The aspect ratio (radial/tangential) of the vascular bundle shape has been shown to correlate with density, which also correlates with the instantaneous strain when stress is applied [22].

Bamboo has different distribution patterns of vascular bundles among the internodes of a culm [13]. The morphology of vascular bundles may also show different patterns not only in transverse section but among different internodes of a culm. Quantification of detailed distribution and morphological changes of vascular bundles in a culm would provide essential insights to reveal the variation in the physiological functions as well as in the mechanical properties of the bamboo culm. To quantitatively analyze the morphology of vascular bundles, new tools are needed.

Many studies have analyzed of computer visions of wood tissues to classify or identify a species from cross-sectional images [23–30]. Classification of bamboo species using machine learning has also been performed [31]. Extraction of vascular bundles and fibers from cross-sectional images of bamboo has been reported using the YOLO algorithm [32] and a K-means clustering algorithm [19]; however, comprehensive morphological quantification of vascular bundles is still lacking.

The focus is to quantify the morphological differences among the various internodes which are most likely to influence the mechanical and physiological properties of bamboo culms. The present study used the U-Net model to construct a highly accurate vascular bundle extraction model from a relatively small number of training images. A robust U-Net model generated from images of different internodes revealed significant changes in vascular bundle distribution and morphology among internodes in culms. Furthermore, the variational autoencoder model successfully estimated continuous morphological transformation of the vascular bundles in the radial direction.

## Materials and methods

### Plant materials

**Fig 1:**
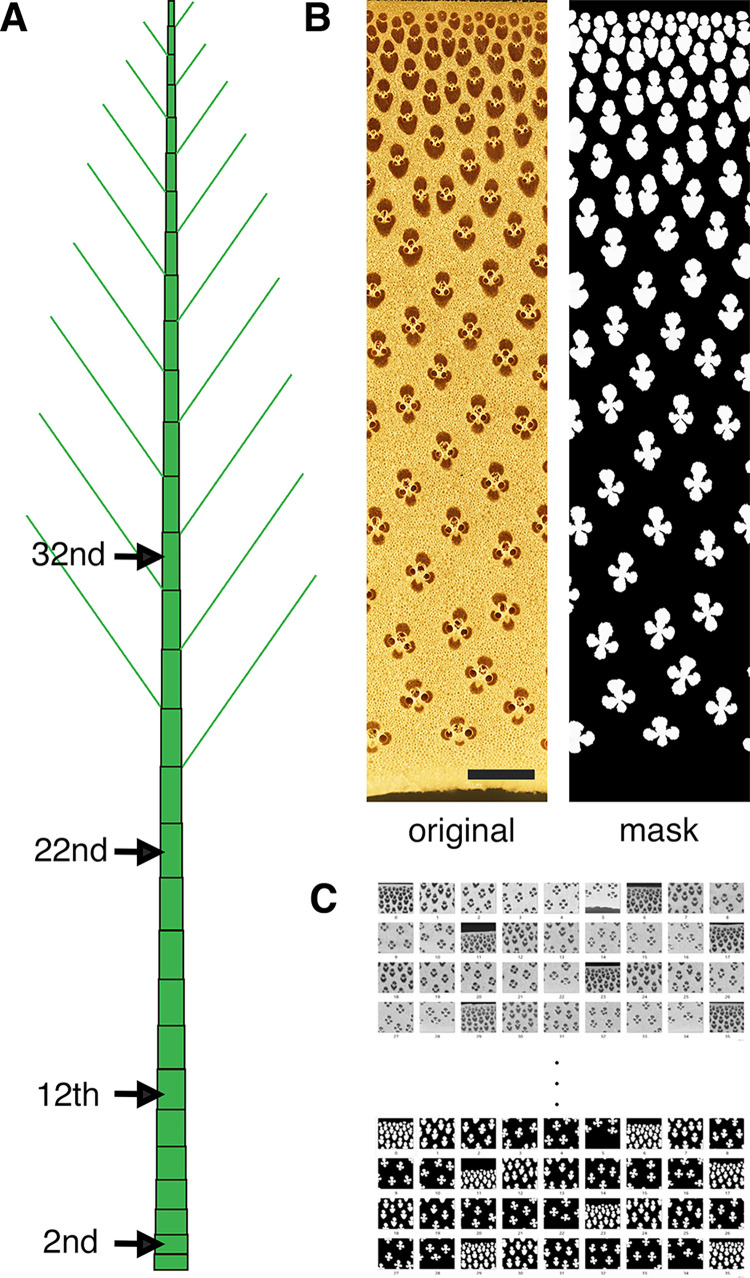
Preparation of training data for extracting vascular bundles from cross section images. Different internodes (the 2nd, 12th, 22nd, and 32nd internodes) in culms (A) were used to prepare blocks and cross sectional images were obtained (B, left side), by which mask images were drawn by hand (B, right side). Scale = 1 mm. Original images and mask images pairs were cropped with gray scale (C) to train U-Net model.

Moso bamboo (*Phyllostachys pubescens*) culms of 1–5 years old (**Tables 1** and **2**) were sampled at a site in Nobeoka, Miyazaki, Japan. The lowermost internode above the ground was designated 0, and then internodes were numbered sequentially from the base to the top of the culm. The 2nd, 12th, 22nd, and 32nd internodes were collected (**Fig 1**).

**Table 1:** Samples used in the present study for training U-Net models.

Model name	Culm	Internode	Block
Model 1	A (1 year old)	2nd	22
Model 2	A (1 year old)	2nd	26
	B (5 year old)	12th	3
	B (5 year old)	22nd	3
	C (4 year old)	32nd	1
	B (5 year old)	32nd	2

**Table 2:** Test images used in the present study to analyze by Model 2.

Culm	Internode	Block
A (1 year old)	2nd	3
	12th	3
	22nd	3
	32nd	3
D (2-year-old)	2nd	3
C (4-year-old)	2nd	3
	12th	3
	22nd	3
	32nd	3
B (5-year-old)	12th	3
	22nd	3
	32nd	3

A combined image containing epidermis to pith with the same tangential width was obtained from each block.

### Training U-Net model

Segmentation of vascular bundles was performed following the U-Net model proposed by Ronneberger et al. [33]. Blocks of 5–10 mm tangentially and 20 mm axially were made from each internode to include all tissue in the radial direction. Transverse sections of blocks were smoothed with a sliding microtome and photographed under an optical microscope with a 4 × objective lens with a resolution of 2.35 μm/pixel. The images were then combined to produce a continuous transverse image from the epidermis to the pith cavity. Mask images were prepared to show the vascular bundle region of the transverse sectional image. The type and number of blocks used for preparation of mask images are shown in **Table 1**.

The mask images for training set were prepared manually from the original images to size as 512 × 512 pixels with the resolution of 4.70 μm/pixel in grayscale. The training sets were created for two U-Net models: 130 sets of cropped images from the 2nd internode were trained on a U-Net model (Model 1), and 264 sets of cropped images from the 2nd, 12th, 22nd, 32nd internodes were trained on a second U-Net model (Model 2) (**Table 1**). The accuracy, the matching of the predicted area and the training mask were used for the measure of the U-Net models.

The U-Net models created were applied to the test images, and the vascular bundle predictive image was labeled. Various parameters of labeled vascular bundles were calculated using a scikit-image region props module. The centroid of the labeled vascular bundle was used to express the position of vascular bundle from the epidermis.

### Variable autoencoder model for prediction of vascular bundle morphogenesis

Variational autoencoder (VAE) is a type of generative model that compresses image information to the latent space and regenerates this information to restore the original image. In between two convolutional networks—decoder and encoder—a latent space exists where the image information is dimensionally reduced. The VAE is a model that uses probability distributions, which means that unknown data can be created probabilistically using a model obtained from training data [34]. The dimension of the latent space was set to 200 vectors, following the model of Foster [35].

A total of 2024 images of extracted vascular bundles were sized as 256 × 256 pixels and used as the training dataset. A model was created after training in 5000 epochs and was used to generate a “morphing” as an attempt to simulate the differentiation stage of the vascular bundles. Morphing was generated by selecting and connecting 5 points in the latent space of vascular bundles selected from the relative radial position.

### Statistical analysis

Statistical significance was tested using one-way ANOVA followed by a post-hoc Tukey test or Kruskal-Wallis test followed by DSCF test using a scikit-posthocs module.

## Results

### Extraction of vascular bundles from transverse sectional images by U-Net models

**Fig 2:**
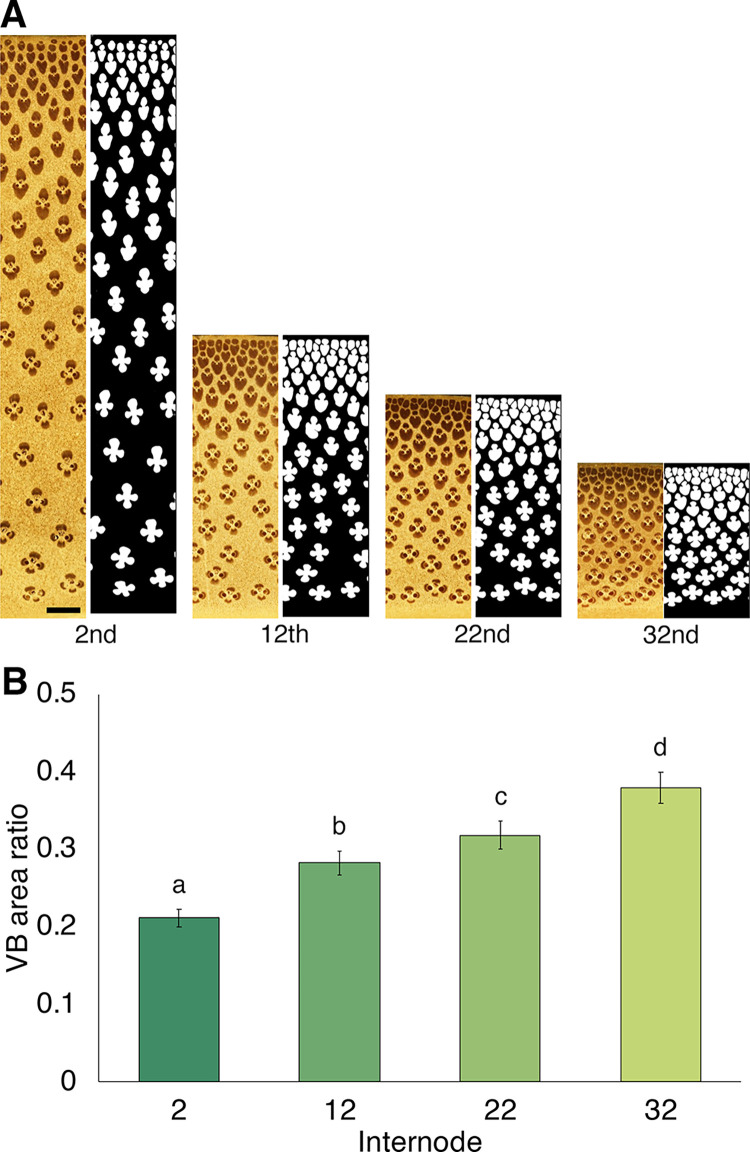
Characteristics of different internodes used for this study. (A) original (left) and labeled (right) images obtained by Model 2. Scale = 1 mm. (B) area ratio of vascular bundles in each culm. Data are mean ± SD (*n* = 9) from three different culms. Different characters indicate significant differences (*p* < 0.05) by Tukey’s test.

To create mask images, transverse sectional images were obtained from 22 blocks of the 2nd internode, which was close to the base. These images were divided into 512 × 512 pixels, and the obtained 130 sets of images were used to train the U-Net model (Model 1), resulting in a minimum loss of 0.04 and an accuracy rate of 98%. We first considered that the second internode was appropriate to model because of the variety of vascular bundles’ shapes seen, but the obtained model was not sufficiently robust to extract vascular bundles from other internodes (**S1 Table**). To extract vascular bundles from different internodes, a robust vascular bundle extraction model (Model 2) was created by further addition of different training images also obtained from internodes other than the 2nd internode (**Table 1**). Model 2 had a minimum loss of 0.04 and an accuracy of 98% (**S1A Fig**). Compared with the predicted images from Model 1, errors of the upper internodes were remarkably decreased (**S1 Table**). In the labeled images predicted by Model 1, vascular bundles, especially those near the epidermis, were incorrectly connected, whereas such errors were greatly reduced in the labeled images predicted by Model 2 (**Fig 2A**, **S1B Fig**). These results indicate that Model 2 is better for recognition of vascular bundles in various internodes and imply that the distribution and morphology of vascular bundles differ between the lower and upper parts of the culm. Calculation of the area ratio of vascular bundle in each internode predicted by Model 2 revealed that the area ratio of vascular bundle was significantly higher in the upper internodes (**Fig 2B**). This result provides quantitative evidence that supports findings of a previous report [22].

### Distribution and morphological patterns of vascular bundles in the radial direction

**Fig 3:**
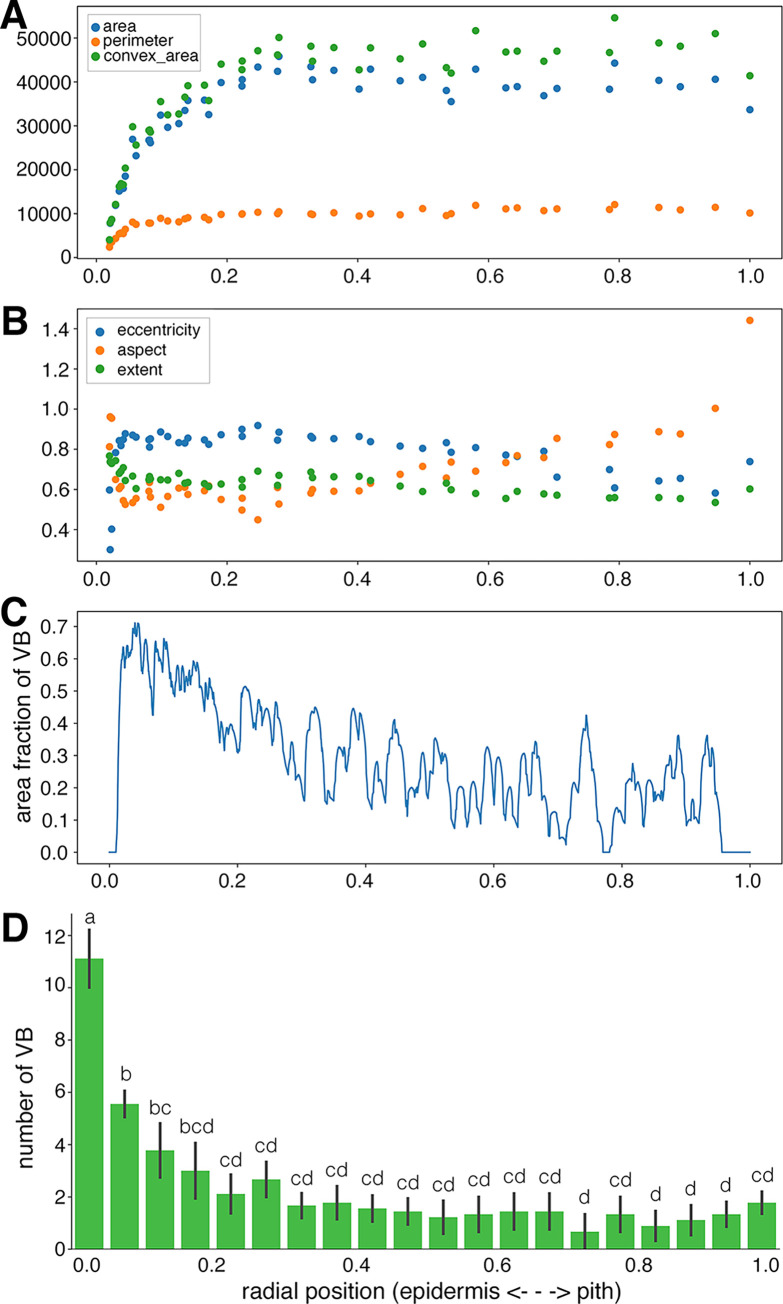
Analyses example of vascular bundles of the 2nd internode extracted by U-Net model 2 (A-C). Radial position is relative position from epidermis (0) to pith cavity (1). (A) area (blue), perimeter (orange), convex area (green); (B) eccentricity (blue), aspect (tangential width/ radial width, orange), extent (vascular bundle area/area of bounding rectangle of vascular bundle, green); (C) area fraction of vascular bundles. D, Number of vascular bundles along with radial direction in the 2nd internode. Data are mean ± SD (*n* = 9) from three different culms. Different characters indicate significant differences (*p* < 0.05) by DSCF test.

Vascular bundles were extracted from the predicted images and analyzed to reveal changes in vascular bundle morphology and distribution in the radial direction (**Fig 3**). Area, perimeter, and convex area showed a similar pattern in which the value was minimum at the epidermis side and increased toward the pith side (**Fig 3A**). Eccentricity sharply increased at epidermis side, and then became almost constant with variation at the pith side. Aspect ratio (tangential width/radial width) sharply decreased at epidermis side showing about 0.5, and then gradually increased as over 1.0 toward pith side. Extent—defined as an area ratio of vascular bundle area versus the bounding rectangle area, reflecting the complexity of vascular bundle morphology—was decreased gradually from the epidermis side to the pith side (**Fig 3B**). The vascular bundle area occupation ratio and number were largest at the epidermis side (**Fig 3C** and 3**D**). These results were consistent with those of a previous report noting the epidermis side has more vascular bundles compared with the pith side [22].

### Distribution and morphological patterns of vascular bundles among different internodes

**Fig 4:**
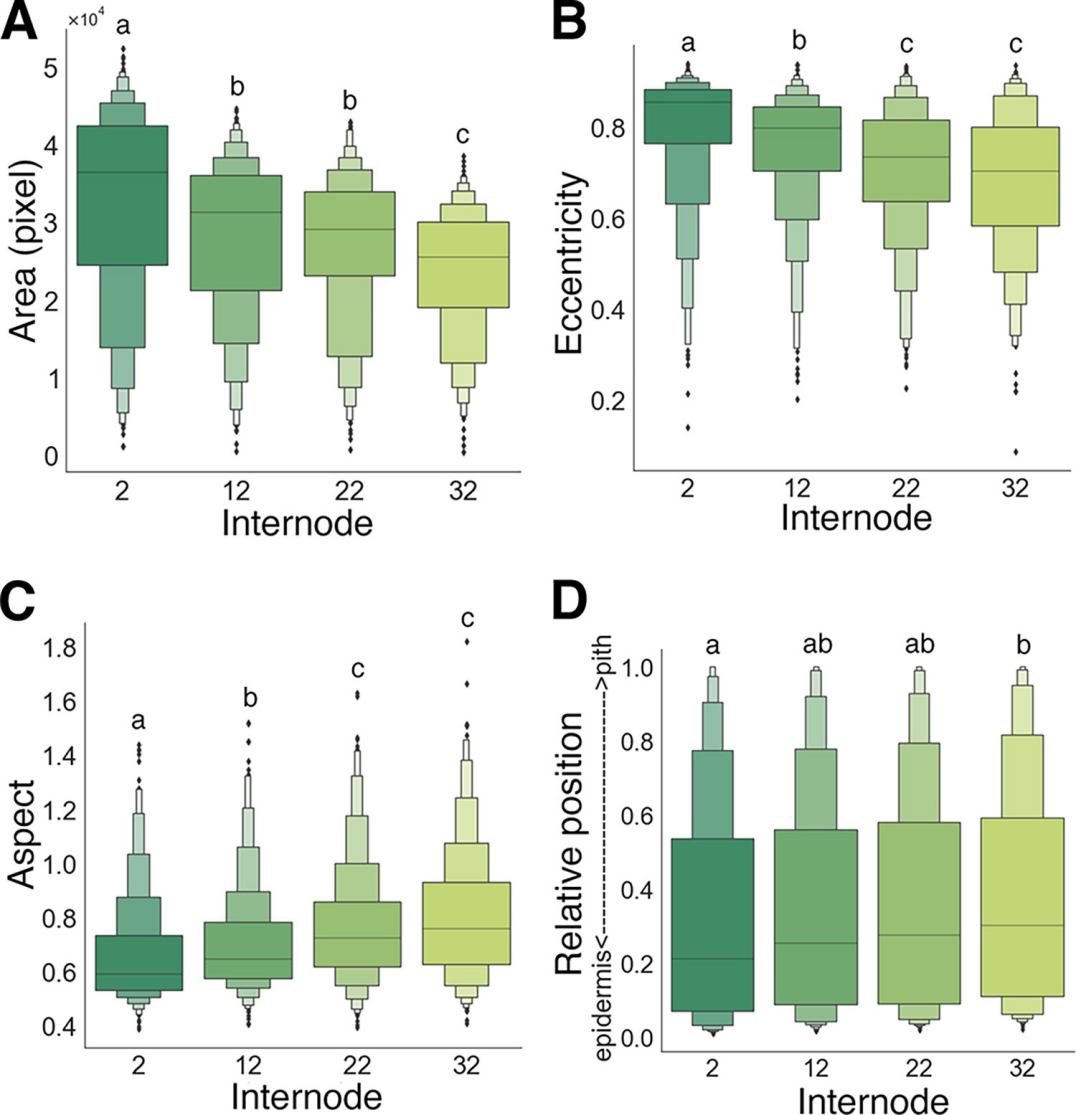
Morphology and distributions of vascular bundles in different internodes. Area (A), eccentricity (B), aspect (C, tangential width/radial width), relative distance (D) of vascular bundles extracted by Model 2. Box plots were made by extracted vascular bundles (*n* ≧ 357) from 9 blocks from 3 culms of different ages (see Table 2). Different letters indicate significant differences (*p* < 0.05, DSCF test).

**Fig 5:**
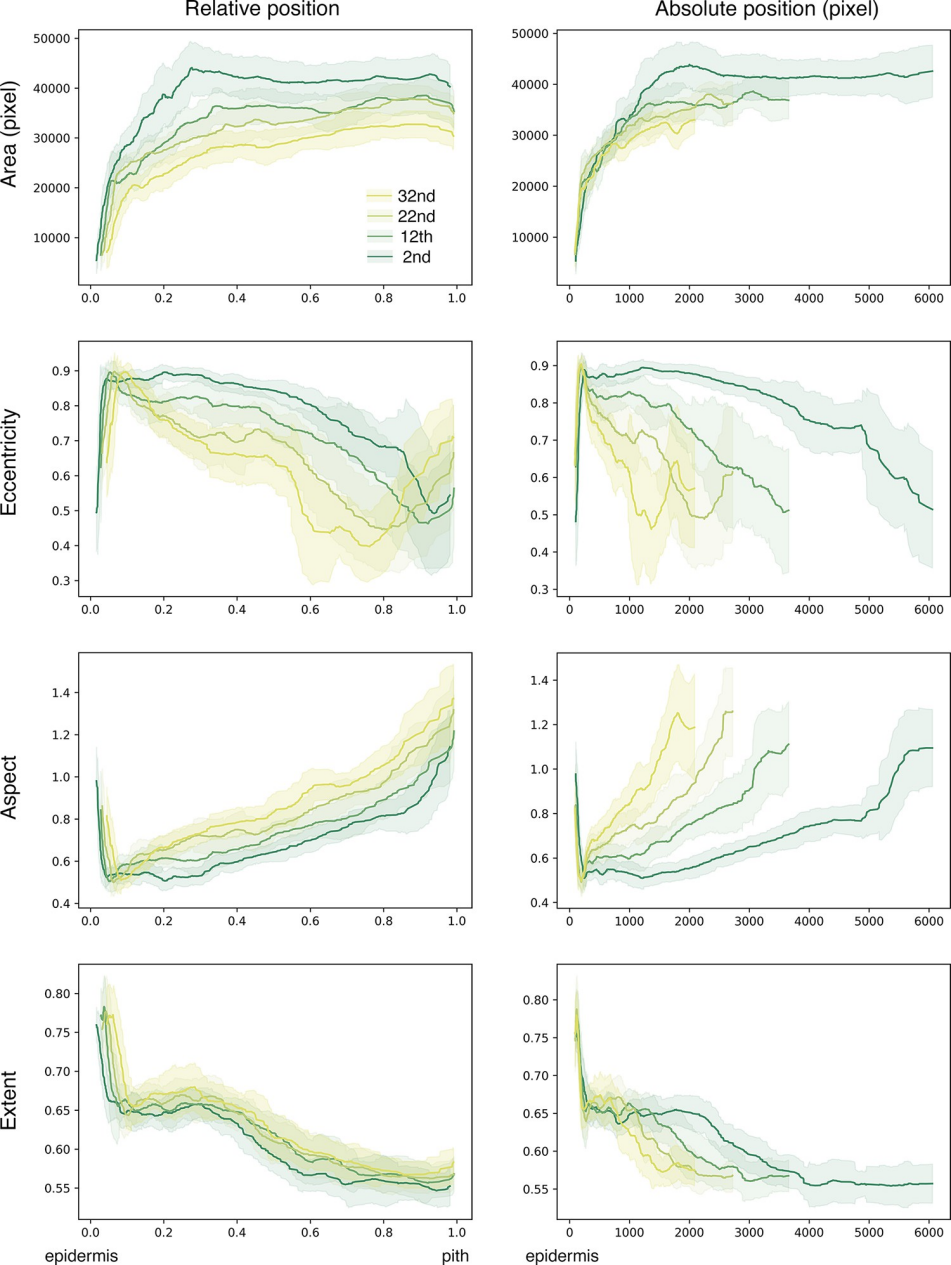
Fluctuations of area, eccentricity, aspect ratio and extent according to relative position (left) or absolute position (right) from epidermis of each internode. Data are represented as moving average ± SD of 25 values obtained from extracted vascular bundles (*n* ≧ 357) from 9 blocks from 3 culms of different ages (see Table 2).

Analysis of vascular bundle morphology revealed significant differences of morphological features among different internodes (**Figs 4** and **5**). The area of vascular bundles was larger in the lower internodes (**Fig 4A**). Eccentricity was also revealed that the lower internodes had a higher proportion of vascular bundles with a shape similar to an ellipse (**Fig 4B**). Moreover, the aspect ratio shows that the lower internodes have more elongated vascular bundles in the radial direction (**Fig 4C**). The lower internodes tended to have more vascular bundles distributed on the epidermis than the upper internodes, with a significant difference between the second and 32nd internodes (**Fig 4D**). In the present study, the extent showed no significant difference among internodes (**Fig 5**).

**Fig 5** shows fluctuations of each parameter according to the radial position in different internodes. Fluctuations of area, aspect ratio, and extent of vascular bundles by their relative position were similar among different internodes, whereas fluctuations of eccentricity were different between the lower internodes and upper internodes. Radial directional fluctuations were associated with an absolute position from the epidermis, which clearly showed that the fluctuations of each parameter were identical among different internodes at the epidermis side, whereas the fluctuations of each parameter were different between internodes in regions by more than approximately 200 pixels (for eccentricity and aspect) or approximately 800 pixels (for area and extent) apart from the epidermis.

### Morphological transformation of vascular bundles in the radial direction by a VAE model

**Fig 6:**
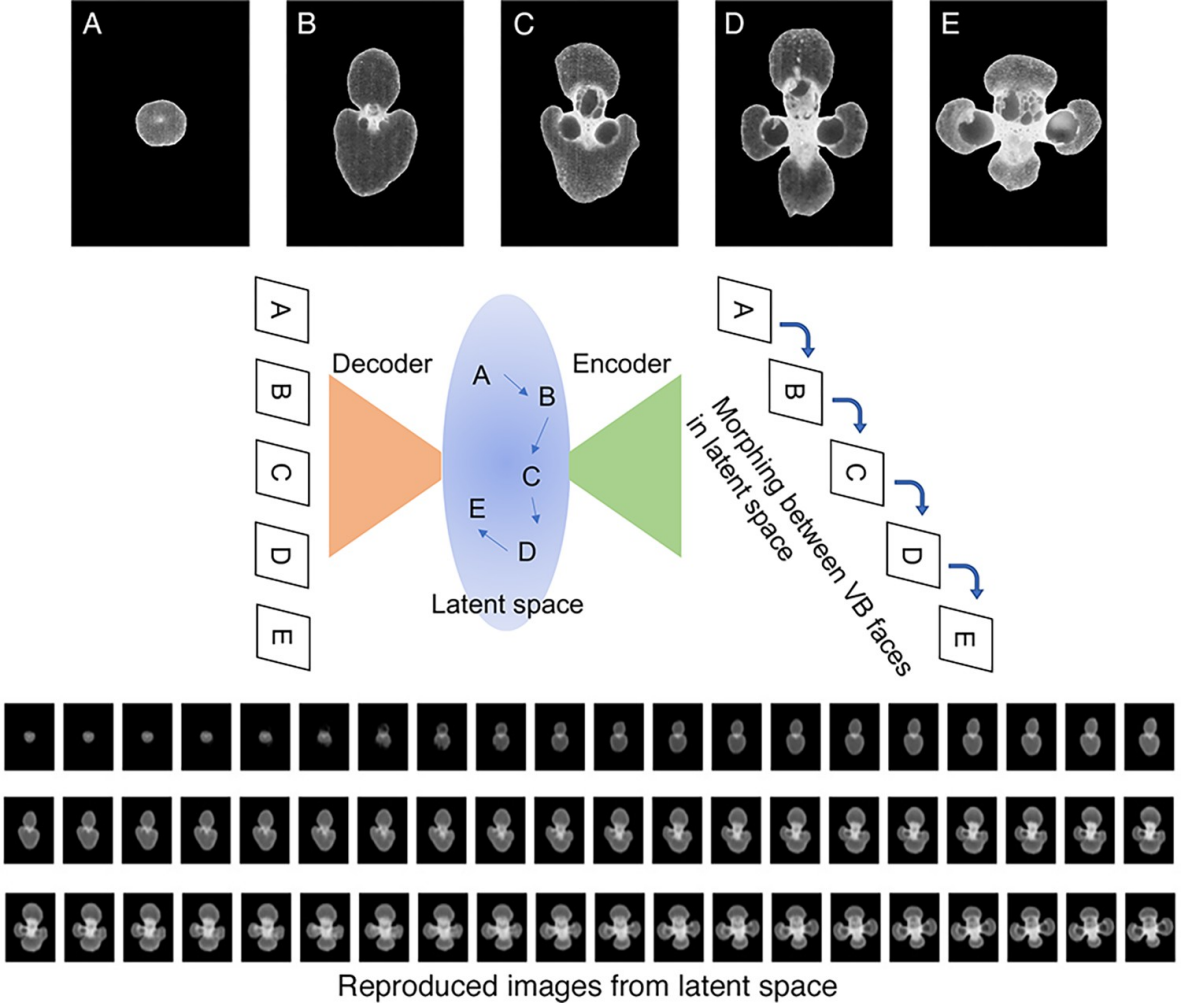
Variable autoencoder model made from extracted vascular bundles by Model 1. Specific extracted vascular bundles (relative position of A = 0.01, B = 0.09, C = 0.40, D = 0.71, E = 1.00) were used for generating specific vectors in latent space.

Each parameter can reveal each facet of the feature of vascular bundles; however, many of other morphological features cannot be described by such parameters. Thus, we tried generation of the best fit model to predict the continuous change of vascular bundle morphology from the epidermis toward the pith side. The morphological transformation in vascular bundles from the epidermis toward the pith were regarded as the development of vascular bundles, and the continuous changes in morphology of vascular bundles were expressed as vectorial changes by a VAE model (**Fig 6**). A VAE model was trained using vascular bundles extracted by Model 1 from all relative radial positions. The morphing created clearly described morphological transformation along the radial direction (**S1 Movie**). At first, development of vascular bundle fibers was observed, followed by development of phloem and xylem inside of the vascular bundles to divide the vascular bundle fibers into two regions. As the diameters of metaxylems enlarged, vascular bundle fibers were divided further into four regions. Finally, on the pith side, the metaxylems were the most distant and the aspect ratio (tangential/radial) of the vascular bundles was largest.

The morphing movie was created to show morphological transformation of vascular bundles along the radial direction from the epidermis to the pith side.

## Discussion

Bamboo vascular bundles appear to be distributed with a certain regularity in the radial direction, which may contribute to the mechanical strength and physiological functions of bamboo. The area fraction of the vascular bundle reached as high as 70% at the epidermal side (**Fig 3**), which was greater than that noted in a previous report [22]. This finding may be due to the fact that the present pixelwise analysis on higher resolution images revealed more detailed information than previous reports. The concentration of vascular bundles on the epidermal side can produce mechanical strength more effectively than uniform distribution [15]. The vascular bundles consisting mainly of vascular fibers on the epidermal side (**Fig 6**, **S1 Movie**) may help to further enhance this effect. Kanzawa et al. showed that the aspect ratio (radial width/tangential width) was smaller on the pith side than on the epidermal side, which was correlated with density [22]; a similar trend was observed in the present study (**Fig 5**). A single row of vascular bundles in the tangential direction was observed at the most pith side (**Fig 2A**), reflecting an increased frequency of vascular bundles at the pith side (**Fig 3D**). These vascular bundles at the most pith side showed ellipses with a long axis in tangential direction (**Figs 5** and **6, S1 Movie**). Such morphology and distribution of vascular bundles on the most pith side may be affected by the formation of the pith cavity.

Bamboo has efficient structure to maintain its plant body with the internode length pattern in a culm [6] or the fiber distribution pattern in the culm wall [15]. The mechanical properties of the upper and lower internodes of a culm show different tendencies [36], which may also be reflected in the distribution and morphology of vascular bundles. The present study clearly demonstrated that the vascular bundle distribution tended to be more epidermal in the internodes closer to the ground, especially with a significant difference between the 2nd internode and the 32nd internode (**Fig 4D**). It is likely that the elucidated difference of the distribution contributes to an increase in the cross-sectional secondary moment and higher bending stiffness in the lower internode with less area fraction of the vascular bundle (**Fig 2**), as proposed by a theoretical model study [15].

**Fig 7:**
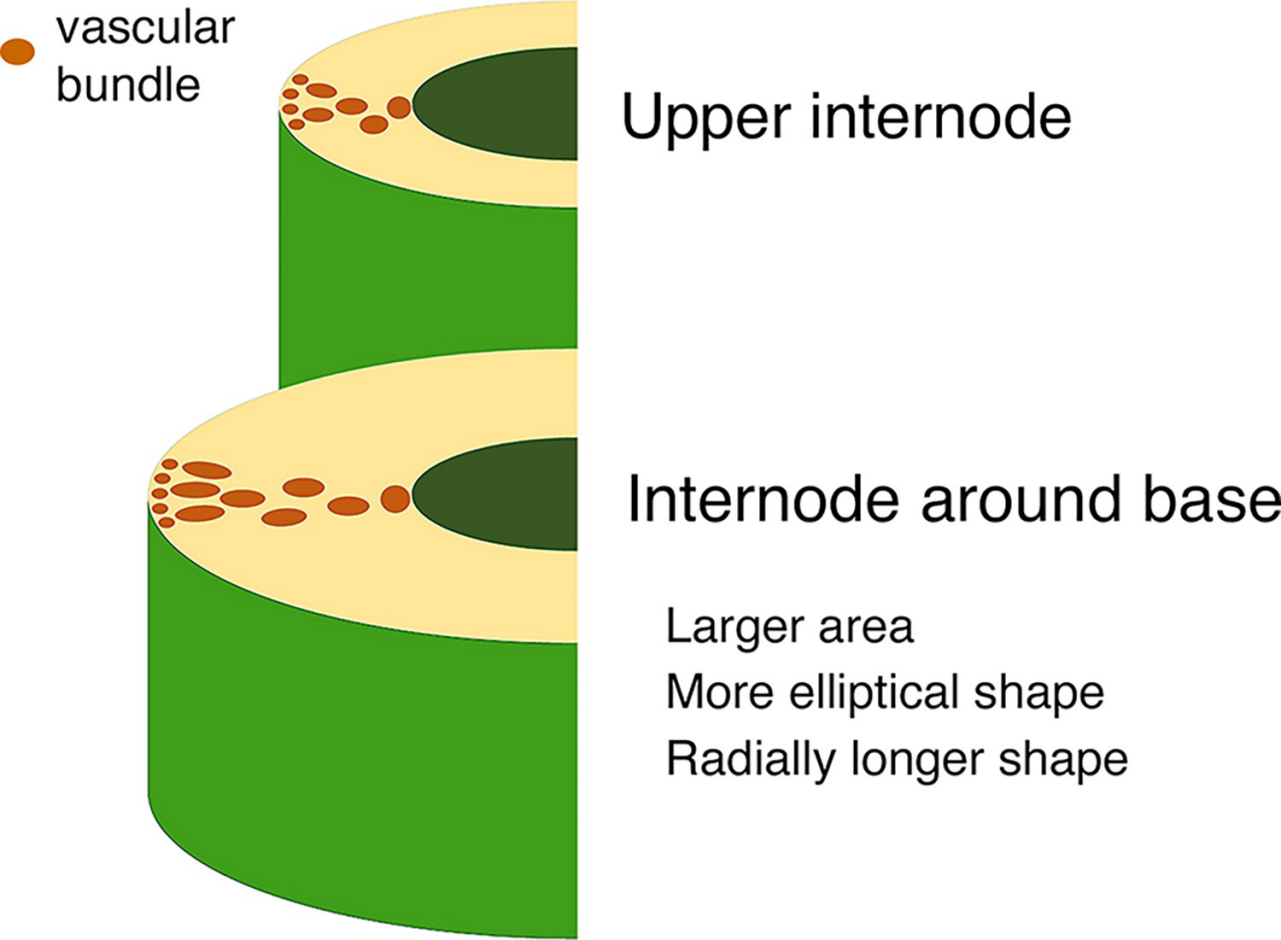
The model of morphological and distribution variance of vascular bundles among internodes in a culm of moso bamboo. Vascular bundles are expressed as brown area. The internode around base has vascular bundles with larger area, more elliptical shape and significantly radially longer morphology than those in upper internodes.

The present quantitative analyses clearly showed that the lower internode contains vascular bundles with significantly larger areas, more elliptical shapes, and significantly radially longer morphologies **(Figs 4** and **5**). Regarding the morphology of vascular bundles, Kanzawa et al. reported that vascular bundles in the upper internodes were radially longer in the outer part of the transverse section than those in middle and lower internodes [22]. In the present study, a similar result was observed only in the limited region, at the outer part of the transverse section (within 10% area from the outermost surface), by the precise analyses (**Fig 5**). The aspect ratio in most parts of the transverse section showed that the lower internode had more vascular bundles with long axes in the radial direction (**Figs 4C and 5**). Such characteristic morphology of vascular bundles in lower internode—specifically, larger areas and more elliptical shapes in the radial direction, as well as the vascular bundle distribution and thick culm wall (**Fig 7**)—would contribute to effective mechanical support of the culm by producing greater cross-sectional secondary moments for external forces in the radial direction with a lower area fraction of vascular bundles (**Fig 2**). The U-Net models created in the present study could be helpful for future study to reveal the contribution of a significant difference of vascular bundle morphological characteristics to mechanical properties.

Changes in vascular bundle morphology as well as the distribution between the epidermis and pith side of cross-sectional images (**Figs 3**, **5**, **and 6**) and between the lower and upper parts of the culm (**Figs 4** and **5**) would be related not only to mechanical performance but also to physiological functions. Vascular bundles on the epidermal side have more vascular bundle fibers (**Fig 6**) and thus would be suitable for mechanical support. On the other hand, vascular bundles on the pith side, with larger meta xylems and phloem, would be suitable for efficient water and nutrient transport. The presence of larger diameter of meta vessels and phloem on the pith side than the epidermal side could be a more appropriate arrangement to protect the vessels and phloem from drying or damage by microbes and pests. Further quantitative analyses of each tissues would provide more insights on differences in the physiological functions in a bamboo culm.

Precise measurement using vascular bundles extracted by a deep learning model revealed that changes of several morphological parameters near the epidermis were identical among different internodes, whereas toward the pith side these parameters varied among different internodes (**Fig 5**). These results imply that the morphogenesis mechanism of vascular bundles on the most epidermal side could be common in a culm, whereas that in other parts may differ among different internodes in a culm. The VAE model generated in the present study clearly demonstrated transformation of vascular bundles (**Fig 6**, **S1 Movie**), inspiring us further applications for the estimation of morphological development from fragmented images of several developmental stages. Generative deep learning models could be effective to obtain insights on tissue formation mechanisms not only in bamboo, but also in a variety of other organisms.

## Conclusion

In the present study we achieved to establish generative deep learning models for quantitatively evaluating morphological transformation of vascular bundles in moso bamboo. Our new tools elucidated significant differences in distribution and morphology among vascular bundles in different internodes. Further studies using these tools would provide understanding on the physiological functions as well as in the mechanical properties of the bamboo culm.

## Supporting information

S1 Dataset All raw data of properties of vascular bundles predicted by Model 2. (XLSX)

S1 Fig Evaluation of U-Net models. (PDF)

S1 Movie A movie of figures created by the VAE model. (MP4)

S1 Table Number of errors of connecting vascular bundles extracted by each U-Net model. (PDF)
