# Supplementary material for: Quantitative morphological transformation of vascular bundles in the culm of moso bamboo (Phyllostachys pubescens)
Source: PLoS One. 2023 Sep 21;18(9):e0290732. doi: 10.1371/journal.pone.0290732 (PMC10513337; doi:10.1371/journal.pone.0290732)
Supplement: S1 Fig — (PDF) [file pone.0290732.s002.pdf]

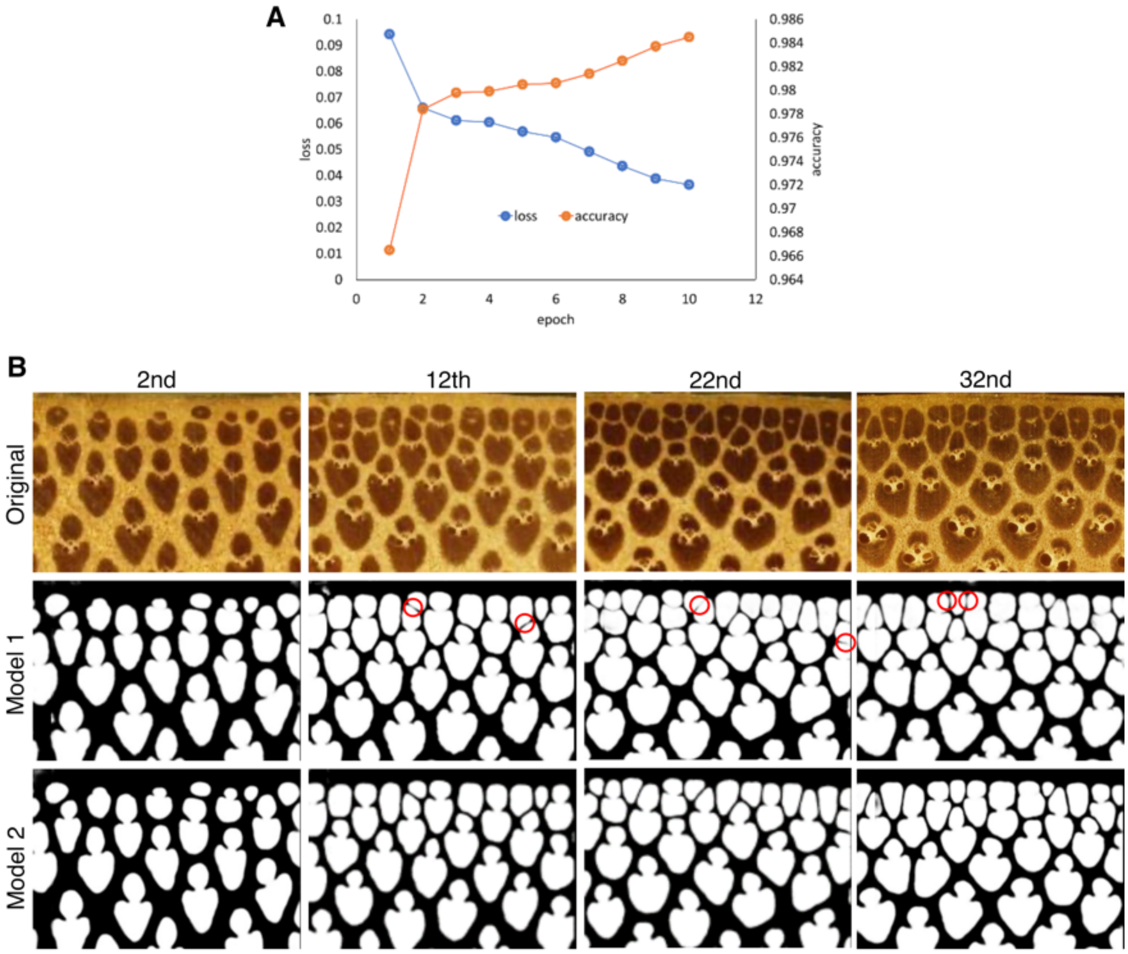

Fig. S1 Evaluation of U-Net Models. A, Training curve of Model 2. B, Comparison of labeled images by Model 1 and Model 2. Red circles indicate errors connecting vascular bundles, which were resolved in labeled images by Model 2.
