# Supplementary material for: Quantitative morphological transformation of vascular bundles in the culm of moso bamboo (Phyllostachys pubescens)
Source: PLoS One. 2023 Sep 21;18(9):e0290732. doi: 10.1371/journal.pone.0290732 (PMC10513337; doi:10.1371/journal.pone.0290732)
Supplement: S1 Table — (PDF) [file pone.0290732.s004.pdf]

Table S1. Number of errors of connecting vascular bundles extracted by each U-Net model

Internode	Model 1	Model 2
2nd	0	0
12th	8	1
22nd	17	3
32nd	24	2
Total error	49	6
Error ratio	3.3%	0.4%

Information of test images used were described in Table 2. Error examples were shown in Fig. S1
